# *GRIA3* p.Met661Thr variant in a female with developmental epileptic encephalopathy

**DOI:** 10.1038/s41439-023-00232-1

**Published:** 2023-02-02

**Authors:** Satomi Okano, Yoshio Makita, Akie Miyamoto, Genya Taketazu, Kayano Kimura, Ikue Fukuda, Hajime Tanaka, Kumiko Yanagi, Tadashi Kaname

**Affiliations:** 1 Department of Pediatrics, Asahikawa Habilitation Center for Children, Hokkaido, Japan; 2 Department of Genetic Counseling, Asahikawa Medical University Hospital, Hokkaido, Japan; 3 Department of Pediatrics, Asahikawa Kosei Hospital, Hokkaido, Japan; 4 Department of Genome Medicine, National Institute for Child Health and Development, Tokyo, Japan

**Keywords:** Disease genetics, Clinical genetics

## Abstract

The X-linked human glutamate receptor subunit 3 (*GRIA3*) gene (MIM *305915, Xq25) encodes ionotropic α amino-3-hydroxy-5-methyl-4-isoxazole propionate (AMPA)-type glutamate receptor subunit 3, which mediates postsynaptic neurotransmission. Variants in this gene can cause a variety of neurological disorders, primarily reported in male patients. Here, we report a female patient with developmental and epileptic encephalopathy who carries the novel *de novo GRIA3* variant NM_007325.5: c.1982T > C: p.Met661Thr.

Human glutamate receptor subunit 3 (*GRIA3*) encodes subunit 3 (GluA3) of the α-3-hydroxy-5-methyl-4-isoxazole propionic receptor (AMPAR). Four types of isoforms, i.e., GluA1–4, produce diverse combinations of pore-forming transmembrane complexes and play important roles in mediating excitatory synaptic transmission via glutamate and synaptic plasticity, which are closely regulated by alternative splicing of flip and flop isoforms^1,2^. AMPARs, including complexes with GluA3, are broadly distributed in the brain, particularly in the hippocampus, cerebral cortex, and thalamus, regions associated with epileptic activity^3^. *GRIA3* variants cause many neurodevelopmental disorders of various degrees of severity, such as cognitive impairment (MIM *300699)^4^, movement disorders^5^, and epilepsy^6^. Approximately 20 variants have been reported, including balanced translocation, deletion, duplication, and missense variants. Most patients have been men whose mothers were carriers, with only five females reported^7–11^.

The patient was a 13-year-old Japanese girl, the second child of healthy, nonconsanguineous parents with no family history of neurological disorders. Her mother had three miscarriages and one stillbirth. She was delivered via scheduled cesarean section at 38 weeks and 3 days of gestation due to fetal distress, acidemia, and limb hypertonia. Her birth weight was 2648 g (−0.86 SD); her head circumference was 33.5 cm (0.43 SD). She exhibited hypertonia and increased deep tendon reflexes. Epilepsy onset occurred at 3 months of age with tonic and clonic seizures. An electroencephalogram (EEG) showed frequent spikes in the central area, which readily developed into generalized spikes and waves (Fig. 1c). Brain magnetic resonance imaging revealed mild frontal lobe atrophy and slight ventricular enlargement (Fig. 1d). Treatment with carbamazepine and ethosuximide was ineffective. The seizures, initially resistant to drug treatment, were gradually brought under control using lamotrigine, clobazam, levetiracetam, and lacosamide. Seizures occurred several times per month, sometimes in clusters. The patient could control her neck at 4 months and roll over at 9 months of age. However, she was bedridden and had poor speaking ability at one year of age. She now presents with severe developmental delay, scoliosis and hip dislocation. Characteristic facial features are not apparent (Fig. 1a, b).

**Fig. 1:**
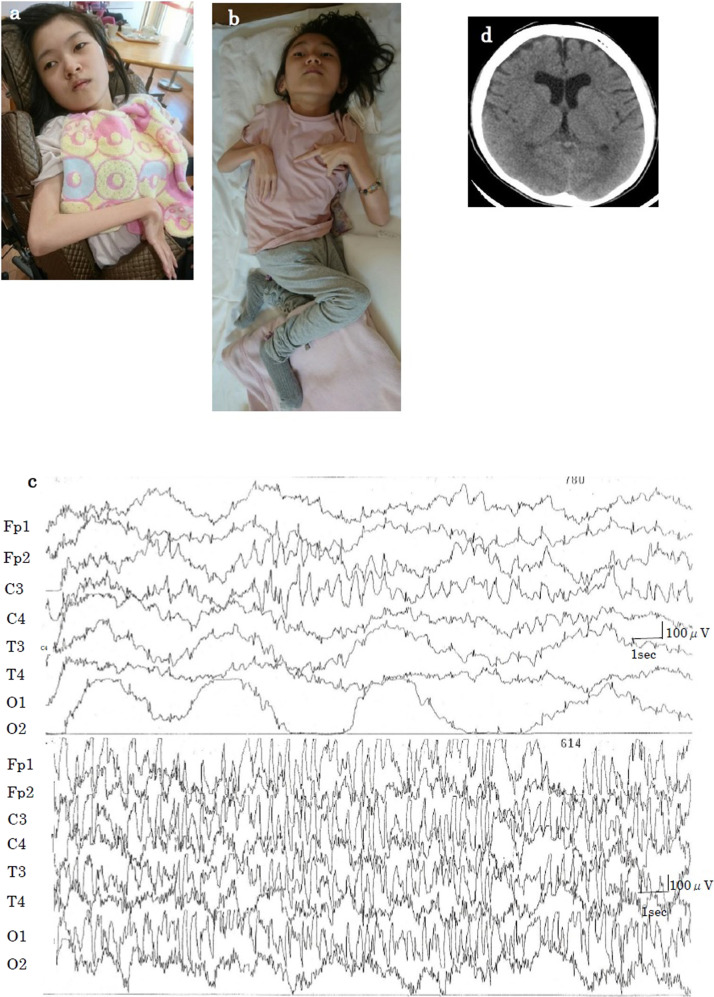
Current features, magnetic resonance imaging scan, and electroencephalogram of the patient. **a**, **b** The patient had no specific facies. She was bedridden, with hypertonia and severe psychomotor delay. **c** Electroencephalogram of the first seizure at 3 months of age. Spikes in the central area that easily developed into generalized waves were noted. **d** Brain magnetic resonance image at 13 years of age. Slight frontal lobe atrophy and ventricular enlargement were not aggravated.

The *FOXG1* variant, analyzed by Sanger sequencing, was not detected during her infancy. Written informed consent for genetic analysis was obtained from her parents; the Ethical Committee of the Asahikawa Habilitation Center for Children (No. R04-03) approved the study. Microarray analysis did not show any pathogenic copy number variants. The Initiative on Rare and Undiagnosed Disease, a nationwide consortium of the Japan Agency for Medical Research and Development, performed whole-exome sequencing and variant filtering for pathogenicity in the patient and her parents. We identified a *de novo* heterozygous variant in the patient: NM_007325.5: c.1982T>C: p.Met661Thr of *GRIA3*. Currently, this variant is not listed in public databases, including the Exome Variant Server (http://evs.gs.washington.edu/EVS/), the 1000 Genome Project database (http://www.internationalgenome.org/), dbSNP (http://www.nebi.nlm.gov/SNP), the Genome Aggregation Database (gnomAD; http://gnomad.broadinstitute.org/), and the Human Genetic Variation Database (http://www.hgvd.genome.med.kyoto-u.ac.jp/). According to the American College of Medical Genetics and Genomics guidelines^12^, the variant was classified as likely pathogenic (PS2, PM2, PP2, PP3). It was confirmed as deleterious by SIFT and probably damaging by Polyphen-2 analyses. No additional variants associated with the patient’s clinical features were identified.

This variant is located in the linker region between the third transmembrane domain (M3) and the S2 extracellular domain of the glutamate binding domain (Fig. 2), next to a previously reported variant, p.Arg660Thr^10^, affecting a highly conserved amino acid. The patient in that case was a female with clinical manifestations similar to those of our patient: hypertonia, epileptic encephalopathy, and global developmental delay. Sun et al. revealed by functional analysis that p.Arg660Thr was a gain-of-function variant that slowed deactivation and desensitization kinetics^10^. A male patient with a gain-of-function variant also exhibited muscle hypertonia^13^. Although we did not conduct a functional study, hypertonia may be indicate a gain of function, as AMPAR is also expressed in the lower motor neurons^14^. Neurodevelopmental disorder with or without seizures and gait abnormalities (#617864), an AMPAR-related disorder, features hypertonia or increased startle reflex. However, epilepsy was reported regardless of the patients’ sex. GluA3-knockout mice reportedly exhibit marked EEG changes, suggesting a critical role in the generation of slow cortical oscillation^15^.

**Fig. 2:**
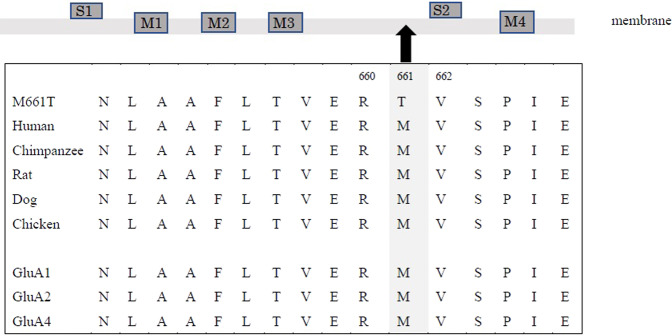
AMPA receptor 3 architecture and sequence alignment. Methionine at position 661 is highly conserved among different species and GluA subunits.

Why *GRIA3* mutations impact female patients remains unknown. Among developmental epileptic encephalopathies, female patients with *WDR45*- or *SMC1A*-related disorders exhibit equal or worse clinical manifestations than male patients^11^. Neither X-inactivation nor mosaicism can entirely account for the range of symptoms or severity. In addition, the association between genotype and phenotype has not been defined, except for the localization of movement disorder variants in the TM4 region^4,5^. Carbamazepine has been reported to be effective for a patient with a gain-of-function variant^13^. In our case, carbamazepine was ineffective, possibly because it does not act directly on AMPAR but inhibits glutamate release from presynaptic neurons^16^. In addition, variants tend not to act in isolation but elicit adaptive changes in other systems, complicating patients’ responsiveness to treatment^17^. Antiepileptic medications that are currently in use may be effective to some extent by manipulating neuronal networks in the brain^18^. We intend to classify antiepileptic medications while taking into account the use of perampanel, a noncompetitive AMPAR antagonist, as a first-line treatment. From the perspective of treatment decisions, the accumulation of case data is crucial.

## HGV database

The relevant data from this Data Report are hosted at the Human Genome Variation Database at 10.6084/m9.figshare.hgv.3276.
